# Quebracho Tannin Bio-Based Adhesives for Plywood

**DOI:** 10.3390/polym14112257

**Published:** 2022-05-31

**Authors:** Johannes Jorda, Emanuele Cesprini, Marius-Cătălin Barbu, Gianluca Tondi, Michela Zanetti, Pavel Král

**Affiliations:** 1 Forest Products Technology and Timber Construction Department, Salzburg University of Applied Sciences, Markt 136a, 5431 Kuchl, Austria; marius.barbu@fh-salzburg.ac.at; 2 Department of Wood Science and Technology, Mendel University, Zemědělská 3, 61300 Brno, Czech Republic; kral@mendelu.cz; 3 Land Environmental Agriculture & Forestry Department, University of Padua, Viale dell’Università 16, 3520 Legnaro, Italy; emanuele.cesprini@studenti.unipd.it (E.C.); gianluca.tondi@unipd.it (G.T.); michela.zanetti@unipd.it (M.Z.); 4 Faculty for Furniture Design and Wood Engineering, Transilvania University of Brasov, B-Dul. Eroilor Nr. 29, 500036 Brasov, Romania

**Keywords:** plywood, quebracho, tannin furfural, biogenic adhesives

## Abstract

Wood-based products are traditionally bonded with synthetic adhesives. Resources availability and ecological concerns have drawn attention to bio-based sources. The use of tannin-based adhesives for engineered wood products has been known for decades, however, these formulations were hardly used for the gluing of solid wood because their rigidity involved low performance. In this work, a completely bio-based formulation consisting of Quebracho (*Schinopsis balancae*) extract and furfural is characterized in terms of viscosity, gel time, and FT-IR spectroscopy. Further, the usability as an adhesive for beech (*Fagus sylvatica*) plywood with regard to press parameters (time and temperature) and its influence on physical (density and thickness) and mechanical properties (modulus of elasticity, modulus of rupture and tensile shear strength) were determined. These polyphenolic adhesives presented non-Newtonian behavior but still good spreading at room temperature as well as evident signs of crosslinking when exposed to 100 °C. Within the press temperature, a range of 125 °C to 140 °C gained suitable results with regard to mechanical properties. The modulus of elasticity of five layered 10 mm beech plywood ranged between 9600 N/mm^2^ and 11,600 N/mm^2^, respectively, with 66 N/mm^2^ to 100 N/mm^2^ for the modulus of rupture. The dry state tensile shear strength of ~2.2 N/mm^2^ matched with other tannin-based formulations, but showed delamination after 24 h of water storage. The proposed quebracho tannin-furfural formulation can be a bio-based alternative adhesive for industrial applicability for special plywood products in a dry environment, and it offers new possibilities in terms of recyclability.

## 1. Introduction

Lignocellulosics are abundant bio-resources, nowadays perceived as a gamechanger in the scope of the climate crisis. Wood products contribute as carbon dioxide (CO_2_) storage sinks due to increasing the time that CO_2_ captured in forests is kept out of the atmosphere. Encouraging more forest growth, wood products enhance the efficiency of forest sinks by acting as carbon stores [1]. Indeed, numerous studies have emphasized the environmental benefits of wood-based materials compared to mineral-based compounds [2,3], due to the low embodied emissions and the lower material intensity of wood [4]. Within wood-based products, wood panels cover a major assortment of applications in the construction, packaging, and furniture sectors [5]. In order to achieve well-distinct properties, wood panel manufacturing adapts the dimensions of engineered wood products (EWPs) through the intelligent (re)assembly of wooden parts. Assembly that is regularly done with the application of adhesive resins. The high market that EWPs are gaining have caused environmental concerns related to the emissions from formaldehyde and other volatile compound that underlie the main adhesives used [6]. Consequently, workable alternatives are required in accordance with environmental standards and safety and market demands to direct future development through a sustainable use of wood. For instance, the generation of adhesives from bio-resources enables both a reduction in the use of chemical reagents harmful to health and a further move away from petroleum derivatives, thus decreasing the carbon footprint of the final product [7]. Actually, recent market forecasts highlight the importance of the bio-adhesives field and a growth from between USD 3.7–6.0 billion in 2020 to USD 5.2–9.7 billion by 2025–2028 is expected [8,9]. Different renewable substances have been proposed as a building block to manufacture bio-resins, from plant protein such as soy, starch based polysaccharides, and lignocellulosic molecules such as lignin and tannins [10,11]. Additionally, to overcome issues related to toxic reagents such as the formaldehyde traditionally used to manufacture wood-based products [12], different bio-based formaldehyde free formulations have been developed. Oktay et al. (2021) used bio-based corn-starch Mimosa tannin sugar adhesives for panels to meet the EN 312:2010 particleboard (P2) standard requirements for interior fittings in a dry state [13]. Similar results are given by Paul et al. (2021) for particleboard bonded with lignin-based adhesives [14]. Plywood, for example, assembled with PVOH–lignin–hexamine showed a dry tensile shear strength of 0.95 N/mm^2^ [15] or with a soybean meal-based adhesive, which displayed excellent water resistance with a tensile shear strength exceeding 1 N/mm^2^ [16]. Ghahri et al. (2022) reported a wet state tensile shear strength of ~0.8 N/mm^2^ for a Quebracho tannin and isolate soy protein adhesive without hardener [17]. According to the mentioned research, tannins are of particular interest due to their chemical structure and good reactivity [18,19], which make these compounds great candidates. Tannins are classified into hydrolysable and condensed, the former class are mixtures of simple phenols, such as pyrogallol and ellagic acid, and esters of glucose, with gallic and digallic acids [20]. The latter, also known as proanthocyanins or flavanol, constitutes more than 90% of world production [20], which due to its reactivity is more suitable for industrial application. Condensed tannins are polyhydroxy-flavan-3-ol oligomers bonded together mostly by C-C bonds between the A rings of the flavanol units and the pyran rings of other flavanol units [19]. Particularly, the polyphenolic structure suggests the comparison and the possible replacement of phenol-formaldehyde (PF) synthetic resins used for gluing EWPs, whose production has seen a sharp increase in the last decade [21]. Moreover, during processing, PF resins have the highest environmental impact of all major synthetic resins [22], ranking tannins as a potential prime substitute.

Deep research has been carried out on the application of tannin adhesives [23,24,25,26]. However, it is useful to mention that a synthetic crosslinker is almost always required to form the three-dimensional polymeric structure. In the current study, an entirely renewable tannin-based adhesive is proposed, using furfural as hardener. Furfural, belonging to the furan compounds, is produced through the acid hydrolysis of biomass [27], and agricultural residues can be used, too [28]. The renewability and the abundance of lignocellulosic biomass make it a viable resource.

This study proposes a new Quebracho tannin-furfural adhesive formulation to be used for the production of plywood. The aim of our investigation is to characterize the Quebracho tannin-furfural adhesive, previously studied and compared with the main synthetic and non-synthetic hardeners [29], in terms of gel time, viscosity, and FT-IR spectroscopy as well as to determine the mechanical performance of five layered beech (*Fagus sylvatica*) plywood with regard to the press parameter of time and temperature, which consequently contribute to the production and the use of bio-based adhesives.

## 2. Materials and Methods

### 2.1. Materials

The tannin-based adhesives were prepared using Quebracho (*Schinopsis balancae*) tannin extract (Fintan 737B), kindly provided by the company Silvateam (S. Michele Mondovì, Cuneo, Italy) and furfural (99%) obtained by International Furan Chemical IFC (Rotterdam, The Netherlands). Sodium hydroxide was purchased from Alfa Aesar (Thermo Fisher, Waltham, MA, USA) and it was applied to change the pH of the formulation.

Pre-conditioned (20 °C, 65% relative air humidity) rotary cut defect free beech (*Fagus sylvatica*) veneers, purchased from Europlac (Topolčany, Slovakia,), with a nominal thickness of 2.2 mm, an average density of 0.72 g/cm^3^, and an average moisture content of 12% were used to prepare the plywood for this study.

### 2.2. Methods

#### 2.2.1. Adhesive Preparation

The tannin-furfural formulation was prepared by mixing under vigorous stirring the commercial extract with water to obtain a 65% homogeneous suspension. The starting pH of 6.7 was adjusted to 8 by adding a 33% sodium hydroxide solution and finally 10% of furfural calculated on solid tannin was added.

#### 2.2.2. Adhesive Characterization

Gel time: 5 g of the formulation were inserted into a glass test tube which was immersed in an oil bath at 100 °C. The transition time to obtain a solid was recorded using a stopwatch. The tests were repeated three times.

Viscosity: A freshly prepared formulation was analyzed with Rheometer Kinexus Lab from Malvern Panalytical (Malvern, UK). The measurement was conducted at 25 °C using cone-shaped geometry spindles with a diameter of 4 cm and a gap between the plates of 0.15 mm. The rotational speed was set from 10 s^−1^ to 300 s^−1^.

FT-IR: A Frontier ATR-FT-MIR from Perkin Elmer (Waltham, MA, USA) was used for scanning the industrial Quebracho powder, Quebracho furfural formulation dried at room temperature for 24 h and the same formulation cross-linked at 100 °C for 24 h. Every spectrum was acquired with the ATR diamond device with 32 scans from 4000 to 600 cm^−1^ and the fingerprint spectral region between 1800 and 600 cm^−1^ was considered after normalization and baseline correction.

#### 2.2.3. Plywood Preparation

The plywood consisted of five layered 90° crosswise oriented 2.2 mm thick beech veneer plies. Adhesive application was carried out manually by weighing the required adhesive amount of 150 g/m^2^ per glue line with a KERN ITB 35K1IP device (Balingen-Frommern, Germany). Pressing was conducted using a Höfler HLOP 280 (Taiskirchen, Austria). Pressure was set to 3 N/mm^2^; press-time was 10 min, 15 min, respectively 20 min and press-temperature was 110 °C, 125 °C and 140 °C.

A pretest to determine the time depended temperature behavior within the glue line during hot pressing as well as in order to check temperature difference between press and glue line was conducted using a Lutron electronic enterprise BTM 4208SD (Taipei City, Taiwan) datalogger with K-couple thermo-wired sensors. The sensors were placed on the outer plies surfaces and within the glue lines between the singular plies. The temperature at the press control unit was adjusted according to the pretest results.

After pressing, the boards were stored until mass constancy under a climate of 20 °C and 65% relative humidity. Test specimen were cut from the plywood boards for the determination of density, bending strength (MOR), stiffness (MOE), and tensile shear strength (TSS).

#### 2.2.4. Plywood Characterization

The density was determined according to EN 323:2005, and it was obtained from the bending test specimen [30].

The density profile was measured with a DENSE-LAB X (EWS, Hammeln, Germany) and the specimen dimensions 50 mm × 50 mm. The thickness was obtained from the bending test specimens. The “Degree of compression” (DoC) was calculated by the percentage based difference between the theoretical thickness of 11 mm of non-compressed veneer ply stack before pressing and the actual thickness of the bending test specimens according to Spulle et al. (2021) [31].

Dry state tensile shear strength (TSS) and 24 h water soaking TSS was determined according to EN 314:2005 with specimen dimensions 100 mm × 25 mm [32]. Modulus of rupture (MOR) and modulus of elasticity (MOE) were determined by a three-point bending test according to EN 310:2005 with specimen dimensions 250 mm × 50 mm [33].

All mechanical properties (SS, MOE, and MOR) were determined using a Zwick/Roell 250 8497.04.00 test device (Ulm, Germany) under constant climatic conditions (rel. humidity 65%, ambient temperature 20 °C). The set-up and the number of specimens of the conducted tests is given in Table 1.

#### 2.2.5. Data Analysis

For statistical evaluation IBM SPSS (Armonk, NY, USA) was used for descriptive data exploration and univariate and multivariate methods for the evaluation of the different Quebracho tannin-furfural bonded plywood test specimen. To determine differences between the press parameters, an ANOVA at a significance level of 95% was used. Multivariate ANOVA was used to determine the influence of “Temperature” and “Press-time” with the “Density” as covariant. The significance of correlations (Pearson) were evaluated using two-sided confidence intervals of 95%.

## 3. Results & Discussion

### 3.1. Adhesive Characterization

**Figure 1:**
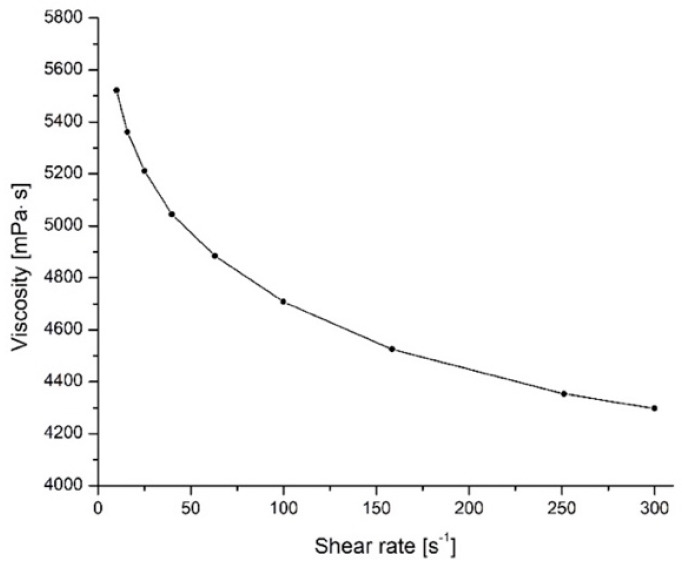
Viscosity of a 65% tannin furfural formulation.

Tannin-furfural adhesives showed the most favorable hardening conditions at pH 8 [29]. Due to the limited viscosity of the adhesive at 50% solid content (s.c.), in this work tannin formulations with 65% s.c. were tested for their viscosity, gel time, and hardening. It was observed that concentrated tannin-furfural formulation presents a non-Newtonian pseudoplastic behavior (Figure 1), described as an increase of shear rate leading to a decrease of viscosity.

In these conditions, the formulation easily resulted in being homogeneously spread on wood. The curing behavior of the formulation was measured through gel time at 100 °C, which was 238 (+/−10) seconds that is slightly slower than commercial urea-formaldehyde’s (UF) as it hardens after 127 s [34] but rather faster than phenol-formaldehyde’s (PF) with a gelation time ranging within 10 min [35].

**Figure 2:**
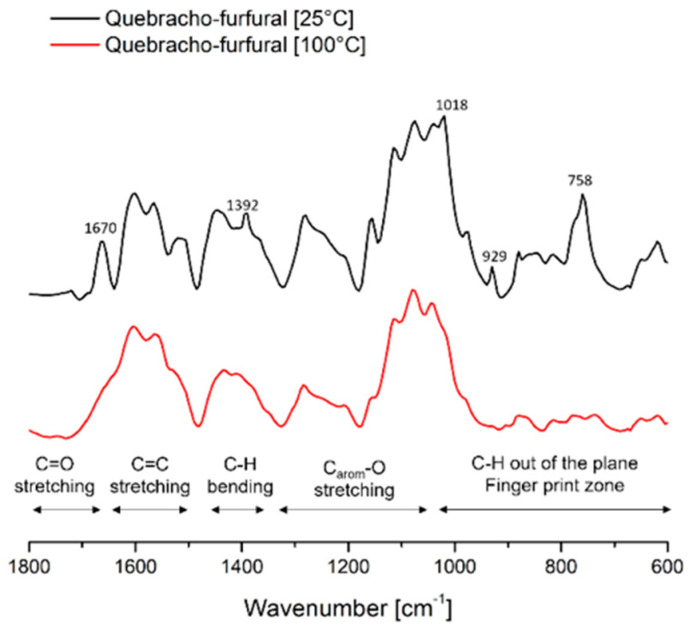
ATR-FTIR of Quebracho tannin furfural formulations at room temperature (black) and cured at 100 °C (red).

From the chemical point of view, the curing process was observed comparing the spectra of the resin exposed 24 h at 25 and 100 °C. Figure 2 reports the spectra of the dry resin before and after curing. Comparing the two spectra, the most evident difference is that after curing the bands become broader suggesting the formation of polymeric structures, in particular the region at lower wavenumber become almost flat due to the steric hindrance for out of plane C-OH wagging and C-H bending vibrations [36,37]. Further major observations are the decreasing/disappearing of some signals such as those at 1670, 1392, 1018, 929, and 758 cm^−1^, which are related to furfural compounds [38,39]. According to these observations, the crosslinking process could be similar to that observed for the polymer with Mimosa extract, involving the bridging through methylene–furanic units [40].

**Figure 3:**
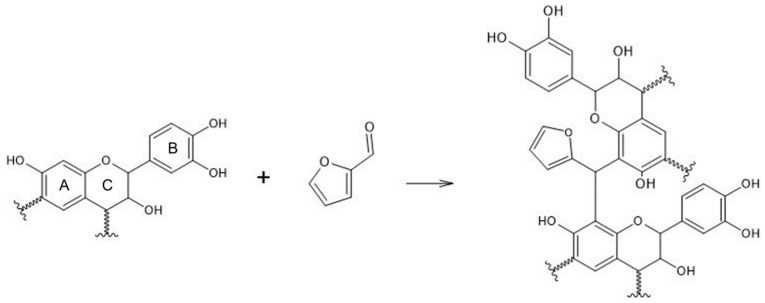
Possible product from Quebracho tannin and furfural reaction.

The main differences between Mimosa and Quebracho tannin is related to the nature of the B ring where the former bonds three hydroxyl groups (pyrogallic unit) [40]. Conversely, the B ring of Quebracho bonds principally two hydroxyl groups (catechol unit) decreasing the reactivity due to the chemistry of phenol [29]. Thus concluding, the reaction between Quebracho and furfural mainly involves the benzene ring A, as reported in Figure 3.

### 3.2. Plywood Characterization

#### 3.2.1. Density

**Figure 4:**
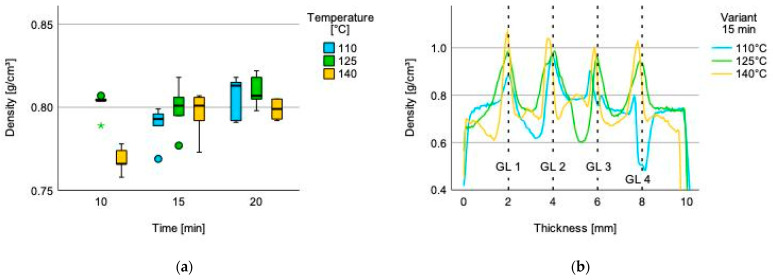
(**a**) Density grouped by time and temperature. Dots and stars within the box plot indicate outliers and (**b**) density profile for the 5-layers plywood glued for 15 min with Quebracho tannin-furfural adhesives at different temperature.

Density is one of the major physical parameters influencing the mechanical properties of plywood while enhancing MOE and tensile strength (TS) [41]. The mean density of the tested groups range between 0.768 g/cm^3^ (press temperature 140 °C; press time 10 min) and 0.810 g/cm^3^ (press temperature 125 °C; press time 20 min) (Figure 4a). The gained results are within the range compared to the values mentioned in the literature for identical five-layered beech plywood set-ups [42,43]. Testing of the density specimen for 110 °C press temperature and 10 min press time was not possible due to delamination after pressing and conditioning.

**Figure 5:**
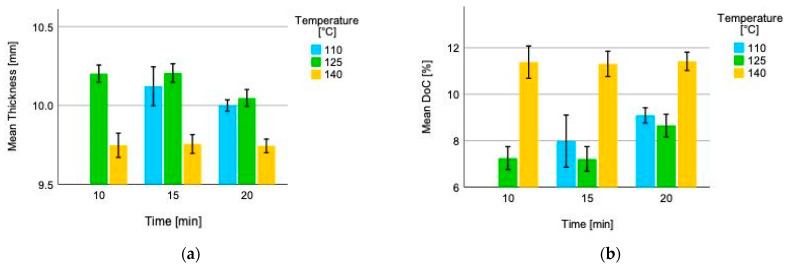
(**a**) Mean thickness and (**b**) mean degree of compression (DoC) of five layered Quebracho tannin furfural bonded plywood. The brackets within the figure indicate the interval ± 1 standard deviation (SD).

The density profile, plotting density against thickness, displays a method to gain information of the bonding performance within the adhesive layer [44,45]. The selected density profiles (Figure 4b) of specimen from the test set 15 min and three different temperatures, demonstrating differing bonding behavior. The specimen for the press temperature of 110 °C reveals delamination within the glue line (GL) 4 due to a significant sharp declined density gap and wider thickness. Further, a double peaking at glue line 3 indicates inappropriate bonding behavior. Test specimen for the press temperature of 125 °C illustrates a deeper adhesive penetration into the plies adjacent to the glue line due to wider and slightly lower density peaks than the selected specimen of the press temperature 140 °C. Compared to the previous described samples, the specimen for 140 °C has a sharper curvature of the density peaks indicating a reduced adhesive penetration into the adjacent wood layers, and a higher degree of compression is visible due to the lower thickness (<10 mm) compared to the other test specimen (>10 mm) with a lower temperature (Figure 5a).

The thickness of the Quebracho tannin-furfural bonded five layered plywood ranges between 9.74 mm (press temperature 140 °C; press time 20 min) and 10.21 mm (press temperature 125 °C; press time 15 min) (Figure 5a), respectively, and between 11.45% and 7.18% for the degree of compression (DoC) (Figure 5b). This is according to Bekhta et al. (2009), stating a compression of ~10% for plywood manufacturing [46].

Thickness and therefore the degree of compression is influenced by the moisture content of veneers, press time and temperature. An elongated press time with a higher temperature influences the chemical wood structure due to a shift toward the glass transition of the singular chemical wood constituents while softening the natural polymeric cellular fiber composite character of wood [47].

#### 3.2.2. Bending Properties

**Figure 6:**
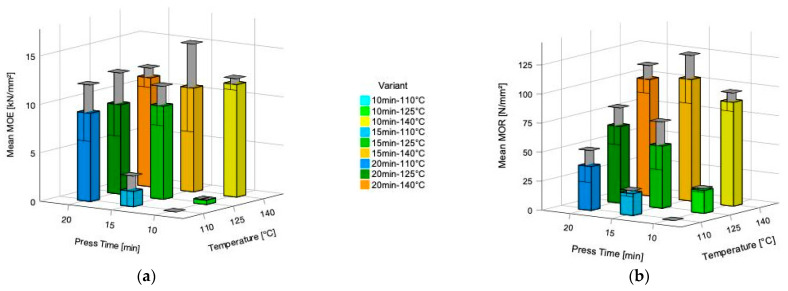
Influence of press time and press temperature of five layered Quebracho Tannin furfural bonded beech plywood on (**a**) Modulus of elasticity (MOE) and (**b**) Modulus of rupture (MOR). The top of the column indicates the means and the bars within the figure represent the standard deviation (SD).

**Figure 7:**
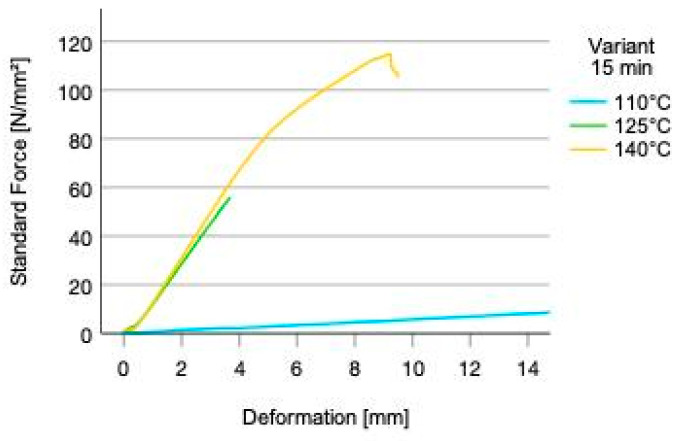
Stress-deformation behavior of selected samples for 15 min press time and three different temperatures.

Modulus of elasticity (MOE) ranges between 448 (SD = 34) N/mm^2^ (press-time 15 min/-temp. 110 °C) and 11,628 (SD = 592) N/mm^2^ (press-time 10 min/-temperature 140 °C) (Figure 6a). Modulus of rupture (MOR) ranges between 18.73 (SD = 2.65) N/mm^2^ (press-time 15 min/-temperature 110 °C) and 104.61 (SD = 20.67) N/mm^2^ (press-time 15 min/-temperature 140 °C) (Figure 6b). Testing of specimen of test-group press time 10 min and press temperature 110 °C could not be carried out due to delamination after pressing and within conditioning. All tested specimen regardless of the test group failed within the adhesive layers, indicating low cohesive strength. Notable is the shift of the failure pattern from the pressure zone to the tension zone of the three-point bending test specimens, with increasing press-temperature and time. This fact reveals an improved adhesive performance with increasing press-time and temperature (Figure 7).

The modulus of elasticity is clearly affected by the combination of temperature and time. At a higher temperature, similar MOE are achieved independently of the pressing time.

Applying 20 min curing time, 110 °C is already sufficient to exceed 9000 N/mm^2^, while at 140 °C with 10 min already a modulus of elasticity exceeding 11,000 N/mm^2^ is reached. Hence, an increase of the temperature above 15 min does not further influence the final MOE. Additionally, the modulus of rupture (MOR) is dependent on the combined effect between temperature and time, where temperature is still crucial (Figure 6b). It can be observed that, the overall preferable pressing conditions for the bending properties require higher temperature (140 °C) and a pressing time of 10 to 15 min.

Comparing the presented MOE and MOR values to the literature, there is a general divergent picture. Niemz (1993) stated general values for MOE between 1500 and 7000 N/mm^2^ for plywood without regard to adhesives [48]. Values for MOE according to DIN 68 705-5 range between 5900 and 9600 N/mm^2^ [49]. Hrazsky and Kral (2005) stated a mean MOE of 12,493 N/mm^2^ and a mean MOR of 77.50 N/mm^2^ for seven layered foiled 10 mm thick beech plywood [50]. Biadala et al. (2020) obtained a mean MOE of 13,720 N/mm^2^ for three layered phenol-formaldehyde bonded beech plywood with a nominal veneer thickness of 1.7 mm and a MOR of 158.4 N/mm^2^ [51]. Lower values are given by Dieste et al. (2008) for MOE with a mean of 9369 N/mm^2^ of *Fagus sylvatica* five layered phenolic resin (150 g/m^2^) bonded plywood at 140 °C press temperature, 10 min of pressing and a pressure of 1 N/mm^2^ [52]. This is 20% lower compared to the presented mean MOE of 11,628 N/mm^2^ for 10 min and 140 °C of the current study. The variation within the numbers can be explained by the natural variation of native wood and its anisotropic behavior. Lohmann (2008) stated for the MOE of *Fagus sylvatica* a range between 10,000 to 18,000 N/mm^2^ and for MOR 74 to 210 N/mm^2^ [53]. Additionally, the mechanical performance of wood-based materials is influenced by the press parameters, according to Réh et al. (2021), as well as the specific lay-up of laminar wood-based products [54]. Further, the type of adhesive has a significant influence on MOE and MOR [55], concluding that the presented adhesive formulation can compete with synthetic phenolic resins in terms of MOE and MOR.

#### 3.2.3. Tensile Shear Strength

**Figure 8:**
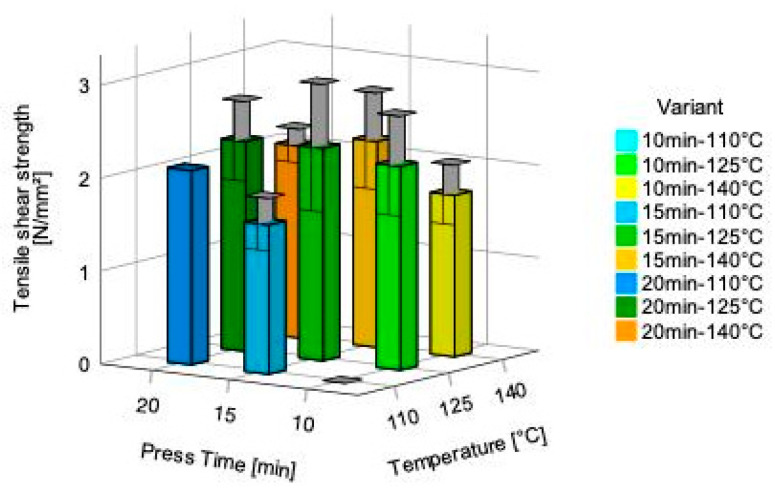
Dry state tensile shear strength for the 5-layered Quebracho tannin bonded plywood. The brackets within the columns of the figure indicate the standard deviation.

Tensile shear strength had been tested in the dry state and after 24 h water storage. The results for the dry state tensile shear strength range between 0.00 N/mm^2^ (press-time 10 min; press-temperature 110 °C), respectively, 1.74 (SD = 0.32) N/mm^2^ (press-time 10 min; press-temperature 140 °C) and 2.29 (SD = 0.69) N/mm^2^ (press-time 15 min; and press-temperature 125 °C) (Figure 8). It has to be noted that specimens of test group 110 °C/10 min failed subsequently before testing due to delamination and only two specimen per test group 125 °C/10 min and test group 110 °C/15 min due to delamination during specimen cutting could be tested. This indicates a poor bonding behavior within the glue line. Tensile shear strength testing at dry state revealed excellent results even at moderate curing temperature (125 °C) with limited influence of the press time.

All tested specimens regardless of the test group failed within the glue line without displaying a wood fracture pattern according to EN 314 [56].

Testing of tensile shear strength for class 1 plywood applications according to EN 314 with 24 h water storage could not be carried out due to delamination failure of all test specimens within the 24 h of immersion into water [32].

Compared to the literature, Xi et al. (2020) gained values for tensile shear strength at a dry state between 0.98 and 1.99 N/mm^2^ for three layered poplar (*Populus tremuloides*) plywood bonded with different Mimosa tannin glucose mixtures [57]. Similar results are stated by Hafiz et al. (2020) for tannin phenol-formaldehyde (TPF) co-polymer bonded rubber wood (*Hevea brasiliensis*) plywood in a range between 1.71 and 2.58 N/mm^2^ and 3.41 N/mm^2^ for the phenol-formaldehyde (PF) bonded reference [58]. Compared to industrial applicated adhesives, Jorda et al. (2021) stated for five layered beech (*Fagus sylvatica*) urea-formaldehyde (UF) bonded plywood a mean tensile shear strength in a dry state of 5.47 N/mm^2^, for melamine-urea formaldehyde (MUF) 6.29 N/mm^2^ and polyurethane (PUR) of 6.74 N/mm^2^ [42]. Biadala et al. (2020) obtained a tensile shear strength value for phenol formaldehyde resin bonded beech plywood after 24 h water soaking of 2.99 N/mm^2^, respectively 2.44 N/mm^2^ after the boiling test [51]. Concluding that the presented Quebracho tannin-furfural adhesive formulation is capable of preserving with other mentioned tannin adhesives formulations for dry state tensile shear strength. Compared to industrial applicated adhesives, the dry-state performance is significantly lower and after 24 h water exposure incapable in terms of water resistance. This could be related to tensions induced by swelling of the singular veneer plies, especially beech (*Fagus sylvatica* L.) reacts sensitive to moisture induced swelling and shrinkage, resulting in low stress transfer capability within the glue line due to the brittle structure of the hardened Quebracho tannin-furfural adhesive. This is in line with several studies mentioning the brittle behavior of tannin-based adhesive formed glue lines [59,60,61].

#### 3.2.4. Statistical Considerations

Significant correlations between thickness and MOE (R = −0.609; *p*-value 0.001), thickness and MOR (R = −0.823; *p*-value 0.001) and the correlation between MOE and MOR (R = 0.831; *p*-value 0.001) could be stated. The correlation of MOE versus density (R = 0.098; *p*-value 0.546) and MOR versus density (R = −0.025; *p*-value 0.876) is not detected. No correlation between tensile shear strength versus MOE (R = −0.147; *p*-value 0.456) and tensile shear strength versus MOR (R = −0.105; *p*-value 0.596) are detected.

The selected press parameters “time” and “temperature” have been accessed by uni- and multivariate methods to determine the influence on density, thickness, modulus of elasticity, modulus of rupture, and tensile shear strength (Table 2).

The one-way ANOVA for the factor “temperature” reveals the influence on density (*p*-value = 0.016; ƞ^2^ 0.202), thickness (*p*-value < 0.001; ƞ^2^ 0.830), modulus of elasticity (*p*-value = 0.001; ƞ^2^ 0.301) and modulus of rupture (*p*-value < 0.001; ƞ^2^ 0.721). It does not influence tensile shear strength (*p*-value = 0.178; ƞ^2^ 0.129). A significant influence can be stated for the factor “time” on density (*p*-value = 0.005; ƞ^2^ 0.246) but not for thickness (*p*-value = 0.425; ƞ^2^ 0.045), tensile shear strength (*p*-value = 0.384; ƞ^2^ 0.074), modulus of elasticity (*p*-value = 0.108; ƞ^2^ 0.113) and modulus of rupture (*p*-value = 0.621; ƞ^2^ 0.025).

The multivariate test conducted for the factors “time” and “temperature” with the covariant “density” displays a similar picture for the factor “temperature” significantly influencing thickness (*p*-value < 0.001), modulus of elasticity (*p*-value < 0.001), modulus of rupture (*p*-value < 0.001) and tensile shear strength (*p*-value 0.048). The factor “time” does significantly influence the thickness (*p*-value < 0.001) but not tensile shear strength (*p*-value 0.127), modulus of elasticity (*p*-value 0.428) and modulus of rupture (*p*-value 0.271).

Comparing the trend of the estimated marginal means trends for temperature, increasing the temperature between 110 °C to 125 °C increases thickness. A further increase in temperature significantly decreases the thickness. This can be explained by the glass transition of the singular chemical constituents of wood resulting in a shape change of the cellular structure [45]. The factor press time displays a similar trend.

Interaction effects between the factors “time” and “temperature” are given for thickness and the mechanical properties of modulus of elasticity and modulus of rupture with a *p*-value < 0.001 but not for tensile shear strength with a *p*-value of 0.303.

For MOE, the “time” has a great influence at a low temperature; but, reaching a temperature between 125 and 140 °C, the increase of pressing time does not lead to improving properties. Similar behavior is found for MOR, but the temperature must reach 140 °C to achieve best features. Tensile shear strength is influenced by time only at 110 °C. With increasing temperature no similar trends are observed, as stated for MOE and MOR.

Concluding the importance of the factor “temperature” on the performance of the mechanical properties led to suggest a temperature range between 125 °C and 140 °C in order to gain sufficient bonding quality. It can be explained by the phenolic character of tannin. The industrial applicated temperature for hot pressing of plywood with PF adhesives is ~130 °C [62].

## 4. Conclusions

The aim of the study was to determine the adhesive characteristics gel time and viscosity as well as the influence of the press parameters, time and temperature, on the selected physical and mechanical properties—density, thickness, modulus of elasticity, modulus of rupture and tensile shear strength—of a totally bio-based sustainable Quebracho tannin-furfural bonded, five-layered beech plywood.

The presented adhesive formulation has shown good viscosity and curing behavior at a relatively low temperature (100 °C), producing polymers after curing. The non-reactivity at room temperature has to be highlighted as a clear advantage in terms of industrial application due to a prolonged open-time and storage duration. Their use as a fully bio-based sustainable adhesive for plywood displayed good bending (modulus of elasticity range ~9600 to ~11,600 N/mm^2^; modulus of rupture range 66 to 100 N/mm^2^) and acceptable tensile shear strength (~2.2 N/mm^2^) in a dry environment, especially for the test specimens in the temperature range 125–140 °C, concluding that the presented formulation is comparable to industrial applicated PF adhesives. Depending on the field of application, as a negative drawback, the low water-resistance due to the brittle character of the adhesive layer structure has to be mentioned as it limits the use of the proposed Quebracho tannin-furfural formulation. On the other hand, it can improve and contribute to recyclability for specific interior plywood applications, as a key element of the bio-based circular economy.

Further research should focus on improving the elastic character of the glue line and enhancing the water resistance of the adhesive, likewise by adding some proportion of isocyanate or epoxy resins in order to further improve the mechanical properties of the adhesive. Additionally, the usability of different wood species, due to the fact that beech (*Fagus sylvatica*) reacts sensitively to moisture induced swelling and shrinkage. Further investigation of press parameters such as pressure and adhesive amount per layer should be taken into consideration. This study used 3 N/mm^2^ as press pressure whereas other studies about tannin-based adhesives range between 1.2 N/mm^2^ [63] and 1.6 N/mm^2^ [64] as well as ~1.4 N/mm^2^ [51] for phenol formaldehyde plywood. For industrial application, the adhesive amount per layer could be further optimized.

## Figures and Tables

**Table 1:** Number of test specimen for the physical and mechanical properties testing of Quebracho tannin-furfural bonded five layered beech plywood.

	Time [min]								
	10			15			200		
Temperature [°C]	110	125	140	110	125	140	110	125	140
	Number of Test Specimens N								
Density	5	5	5	5	5	5	5	5	5
Density profile	5	5	5	5	5	5	5	5	5
Thickness	5	5	5	5	5	5	5	5	5
MOE/MOR	5	5	5	5	5	5	5	5	5
TSS dry state and 24 h	5	5	5	5	5	5	5	5	5

**Table 2:** Results of statistical significance for one-way ANOVA.

Properties	Variable	Mean Square	F-Value	*p*-Value
Density	Temperature	0.001	4.671	0.016 *
	Time	0.001	6.021	0.005 *
Thickness	Temperature	0.654	90.577	<0.001 *
	Time	0.036	0.876	0.425
MOE	Temperature	130,074,609	7.985	0.001 *
	Time	489,227,828.8	2.360	0.108
MOR	Temperature	17,277.397	47.926	<0.001 *
	Time	609.421	0.483	0.621
TSS	Temperature	0.360	1.850	0.178
	Time	0.206	0.994	0.384

* The *p*-value lower than α = 0.05 displays significant influence on the physical and mechanical plywood properties.

## Data Availability

The data presented in this study are available on request from the corresponding author.

## References

[1] Beyer G., Defays M., Fischer M., Fletcher J., de Munck E., de Jaeger F., Van Riet C., Vandeweghe K., Wijnendaele K. (2011). Tackle Clim. Change—Use Wood.

[2] Oliver C.D., Nassar N.T., Lippke B.R., McCarter J.B. (2014). Carbon, Fossil Fuel, and Biodiversity Mitigation with Wood and Forests. J. Sustain. For..

[3] Moncaster A.M., Pomponi F., Symons K.E., Guthrie P.M. (2018). Why Method Matters: Temporal, Spatial and Physical Variations in LCA and Their Impact on Choice of Structural System. Energy Build..

[4] Churkina G., Organschi A., Reyer C.P.O., Ruff A., Vinke K., Liu Z., Reck B.K., Graedel T.E., Schellnhuber H.J. (2020). Buildings as a Global Carbon Sink. Nat. Sustain..

[5] Irle M., Barbu M.C., Thoemen M., Irle M., Sernek M. (2010). Wood-Based Panels: An Introduction for Specialists.

[6] Klarić S., Obučina M. (2020). New Trends in Engineering Wood Technologies. Lect. Notes Netw. Syst..

[7] Heinrich L.A. (2019). Future Opportunities for Bio-Based Adhesives-Advantages beyond Renewability. Green Chem..

[8] Alliedmarketresearch. https://www.alliedmarketresearch.com/bioadhesives-market-A11324.

[9] Marketsandmarkets. https://www.marketsandmarkets.com/Market-Reports/bioadhesive-market-16386893.html.

[10] Hemmilä V., Adamopoulos S., Karlsson O., Kumar A. (2017). Development of Sustainable Bio-Adhesives for Engineered Wood Panels-A Review. RSC Adv..

[11] Ferdosian F., Pan Z., Gao G., Zhao B. (2017). Bio-Based Adhesives and Evaluation for Wood Composites Application. Polymers.

[12] Kristak L., Antov P., Bekhta P., Lubis M.A.R., Iswanto A.H., Reh R., Sedliacik J., Savov V., Taghiyari H.R., Papadopoulos A.N. (2022). Recent Progress in Ultra-Low Formaldehyde Emitting Adhesive Systems and Formaldehyde Scavengers in Wood-Based Panels: A Review. Wood Mater. Sci. Eng..

[13] Oktay S., Kızılcan N., Bengü B. (2021). Development of Bio-Based Cornstarch—Mimosa Tannin—Sugar Adhesive for Interior Particleboard Production. Ind. Crops Prod..

[14] Paul G.B., Timar M.C., Zeleniuc O., Lunguleasa A., Coșereanu C. (2021). Mechanical Properties and Formaldehyde Release of Particleboard Made with Lignin-Based Adhesives. Appl. Sci..

[15] Lubis M.A.R., Labib A., Sudarmanto, Akbar F., Nuryawan A., Antov P., Kristak L., Papadopoulos A.N., Pizzi A. (2022). Influence of Lignin Content and Pressing Time on Plywood Properties Bonded with Cold-Setting Adhesive Based on Poly (Vinyl Alcohol), Lignin, and Hexamine. Polymers.

[16] Zhang Y., Shi R., Xu Y., Chen M., Zhang J., Gao Q., Li J. (2020). Developing a Stable High-Performance Soybean Meal-Based Adhesive Using a Simple High-Pressure Homogenization Technology. J. Clean. Prod..

[17] Ghahri S., Pizzi A., Hajihassani R. (2022). A Study of Concept to Prepare Totally Biosourced Wood Adhesives from Only Soy Protein and Tannin. Polymers.

[18] Pizzi A. (2006). Recent Developments in Eco-Efficient Bio-Based Adhesives for Wood Bonding: Opportunities and Issues. J. Adhes. Sci. Technol..

[19] Shirmohammadli Y., Efhamisisi D., Pizzi A. (2018). Tannins as a Sustainable Raw Material for Green Chemistry: A Review. Ind. Crops Prod..

[20] Pizzi A., Mittal K.L. (2017). Handbook of Adhesive Technology.

[21] Xu Y., Guo L., Zhang H., Zhai H., Ren H. (2019). Research Status, Industrial Application Demand and Prospects of Phenolic Resin. RSC Adv..

[22] Arias A., González-García S., Feijoo G., Moreira M.T. (2021). Tannin-Based Bio-Adhesives for the Wood Panel Industry as Sustainable Alternatives to Petrochemical Resins. J. Ind. Ecol..

[23] Pizzi A. (1979). The Chemistry and Development of Tannin/Urea–Formaldehyde Condensates for Exterior Wood Adhesives. J. Appl. Polym. Sci..

[24] Navarrete P., Pizzi A., Pasch H., Rode K., Delmotte L. (2013). Characterization of Two Maritime Pine Tannins as Wood Adhesives. J. Adhes. Sci. Technol..

[25] Engozogho Anris S.P., Bikoro Bi Athomo A., Safou-Tchiama R., Leroyer L., Vidal M., Charrier B. (2021). Development of Green Adhesives for Fiberboard Manufacturing, Using Okoume Bark Tannins and Hexamine–Characterization by 1H NMR, TMA, TGA and DSC Analysis. J. Adhes. Sci. Technol..

[26] Ballerini A., Despres A., Pizzi A. (2005). Non-Toxic, Zero Emission Tannin-Glyoxal Adhesives for Wood Panels. Holz Roh-Werkst..

[27] Kabbour M., Luque R., Saravanamurugan S., Pandey A., Riisager A. (2020). Furfural as a Platform Chemical: From Production to Applications. Biomass, Biofuels, Biochemicals—Recent Advances in Development of Platform Chemicals.

[28] Bozell J.J., Petersen G.R. (2010). Technology Development for the Production of Biobased Products from Biorefinery Carbohydrates—The US Department of Energy’s “Top 10” Revisited. Green Chem..

[29] Cesprini E., Šket P., Causin V., Zanetti M. (2021). Development of Quebracho (*Schinopsis balansae*) Tannin-Based Thermoset Resins. Polymers.

[30] (2005). Wood-Based Panels—Determination of Density.

[31] Spulle U., Meija A., Kūlinš L., Kopeika E., Liepa K.H., Šillers H., Zudrags K. (2021). Influence of Hot Pressing Technological Parameters on Plywood Bending Properties. BioResources.

[32] (2005). Plywood—Bonding Quality—Test Methods.

[33] (2005). Wood-Based Panels—Determination of Modulus of Elasticity in Bending and of Bending Strength.

[34] Navarrete P., Pizzi A., Tapin-Lingua S., Benjelloun-Mlayah B., Pasch H., Rode K., Delmotte L., Rigolet S. (2012). Low Formaldehyde Emitting Biobased Wood Adhesives Manufactured from Mixtures of Tannin and Glyoxylated Lignin. J. Adhes. Sci. Technol..

[35] Hauptt R.A., Sellers T. (1994). Characterizations of Phenol-Formaldehyde Resol Resins. Ind. Eng. Chem. Res..

[36] Ricci A., Olejar K.J., Parpinello G.P., Kilmartin P.A., Versari A. (2015). Application of Fourier Transform Infrared (FTIR) Spectroscopy in the Characterization of Tannins. Appl. Spectrosc. Rev..

[37] Tondi G., Petutschnigg A. (2015). Middle Infrared (ATR FT-MIR) Characterization of Industrial Tannin Extracts. Ind. Crops Prod..

[38] Mohamad N., Abd-Talib N., Kelly Yong T.L. (2020). Furfural Production from Oil Palm Frond (OPF) under Subcritical Ethanol Conditions. Mater. Today Proc..

[39] Kane S.N., Mishra A., Dutta A.K. (2017). Synthesis of Furfural from Water Hyacinth (*Eichornia croassipes*) This. Mater. Sci. Eng..

[40] Tondi G. (2017). Tannin-Based Copolymer Resins: Synthesis and Characterization by Solid State 13C NMR and FT-IR Spectroscopy. Polymers.

[41] Wagenführ A., Scholz F. (2008). Taschenbuch der Holztechnik.

[42] Jorda J., Kain G., Barbu M.-C., Petutschnigg A., Král P. (2021). Influence of Adhesive Systems on the Mechanical and Physical Properties of Flax Fiber Reinforced Beech Plywood. Polymers.

[43] Jorda J., Kain G., Barbu M.-C., Köll B., Petutschnigg A., Král P. (2022). Mechanical Properties of Cellulose and Flax Fiber Unidirectional Reinforced Plywood. Polymers.

[44] Mansouri H.R., Pizzi A., Leban J.M. (2006). Improved Water Resistance of UF Adhesives for Plywood by Small PMDI Additions. Holz Roh-Werkst..

[45] Luo J., Luo J., Gao Q., Li J. (2015). Effects of Heat Treatment on Wet Shear Strength of Plywood Bonded with Soybean Meal-Based Adhesive. Ind. Crops Prod..

[46] Bekhta P., Hiziroglu S., Shepelyuk O. (2009). Properties of Plywood Manufactured from Compressed Veneer as Building Material. Mater. Des..

[47] Cabral J.P., Kafle B., Subhani M., Reiner J., Ashraf M. (2022). Densification of Timber: A Review on the Process, Material Properties, and Application. J. Wood Sci..

[48] Niemz P. (1993). Physik des Holzes und der Holzwerkstoffe.

[49] (1980). Sperrholz Teil 5—Bau-Furniersperrholz aus Buche.

[50] Hrázský J., Král P. (2005). Assessing the Bending Strength and Modulus of Elasticity in Bending of Exterior Foiled Plywoods in Relation to Their Construction. J. For. Sci..

[51] Biadała T., Czarnecki R., Dukarska D. (2020). Water Resistant Plywood of Increased Elasticity Produced from European Wood Species. Wood Res..

[52] Dieste A., Krause A., Bollmus S., Militz H. (2008). Physical and Mechanical Properties of Plywood Produced with 1.3-Dimethylol-4.5-Dihydroxyethyleneurea (DMDHEU)-Modified Veneers of *Betula* sp. and *Fagus Sylvatica*. Holz Roh-Werkst..

[53] Lohmann U. (2010). Holz Handbuch.

[54] Réh R., Krišťák Ľ., Sedliačik J., Bekhta P., Božiková M., Kunecová D., Vozárová V., Tudor E.M., Antov P., Savov V. (2021). Utilization of Birch Bark as an Eco-Friendly Filler in Urea-Formaldehyde Adhesives for Plywood Manufacturing. Polymers.

[55] Bal B.C., Bektaþ Ý. (2014). Some Mechanical Properties of Plywood Produced from Eucalyptus, Beech, and Poplar Veneer. Maderas. Cienc. Tecnol..

[56] (2005). Plywood—Bonding Quality—Part 2 Requierments.

[57] Xi X., Pizzi A., Frihart C.R., Lorenz L., Gerardin C. (2020). Tannin Plywood Bioadhesives with Non-Volatile Aldehydes Generation by Specific Oxidation of Mono- and Disaccharides. Int. J. Adhes. Adhes..

[58] Hafiz N.L.M., Tahir P.M.D., Hua L.S., Abidin Z.Z., Sabaruddin F.A., Yunus N.M., Abdullah U.H., Abdul Khalil H.P.S. (2020). Curing and Thermal Properties of Co-Polymerized Tannin Phenol-Formaldehyde Resin for Bonding Wood Veneers. J. Mater. Res. Technol..

[59] Pizzi A., Scharfetter H.O. (1978). The Chemistry and Development of Tannin-based Adhesives for Exterior Plywood. J. Appl. Polym. Sci..

[60] Ayla C., Parameswaran N. (1980). Macro- and Microtechnological Studies on Beechwood Panels Bonded with *Pinus Brutia* Bark Tannin. Holz Roh-Werkst..

[61] Ferreira É.D.S., Lelis R.C.C., Brito E.D.O., Iwakiri S. Use of Tannin from *Pinus oocarpa* Bark for Manufacture of Plywood. Proceedings of the LI International Convention of Society of Wood Science and Technology.

[62] Sedliačik J., Bekhta P., Potapova O. (2010). Technology of Low-Temperature Production of Plywood Bonded with Modified Phenol-Formaldehyde Resin. Wood Res..

[63] Moubarik A., Pizzi A., Allal A., Charrier F., Charrier B. (2009). Cornstarch and Tannin in Phenol-Formaldehyde Resins for Plywood Production. Ind. Crops Prod..

[64] Stefani P.M., Peña C., Ruseckaite R.A., Piter J.C., Mondragon I. (2008). Processing Conditions Analysis of *Eucalyptus Globulus* Plywood Bonded with Resol-Tannin Adhesives. Bioresour. Technol..

